# Epidemiological Characteristics of Dengue Disease in Mexico (2014–2025): A Descriptive Analysis of a Hyperendemic Country

**DOI:** 10.3390/pathogens15020190

**Published:** 2026-02-08

**Authors:** Rosa Cremades, Elena Sandoval-Pinto, Ana Maria Ortega-Prieto, Jose M. Jimenez-Guardeño, Héctor Raúl Pérez-Gómez, Juan Carlos Lona Reyes, Erick Sierra-Díaz, Jose Angel Regla-Nava

**Affiliations:** 1 Department of Microbiology and Pathology, University Center for Health Science (CUCS), University of Guadalajara, Guadalajara 44340, Mexico; rosa.cremades@academicos.udg.mx; 2 Department of Cellular and Molecular Biology, University of Guadalajara, Guadalajara 45200, Mexico; elena.sandovalp@academicos.udg.mx; 3 Instituto de Investigación Biomédica de Málaga y Plataforma en Nanomedicina-IBIMA Plataforma BIONAND, 29590 Málaga, Spain; ana.ortega@uma.es (A.M.O.-P.); jose.jimenez@uma.es (J.M.J.-G.); 4 Unidad de Gestión Clínica de Enfermedades Infecciosas, Microbiología y Medicina Preventiva, Hospital Regional Universitario de Málaga, 29010 Málaga, Spain; 5 Departamento de Microbiología, Universidad de Málaga, 29010 Málaga, Spain; 6 Institute of Infectious and Experimental Pathology, University Center of Health Sciences, University of Guadalajara, Guadalajara 44280, Mexico; hector.pgomez@academicos.udg.mx; 7 Pediatric Infectology Service, Hospital Civil de Guadalajara, Guadalajara 44340, Mexico; carloslona5@hotmail.com; 8 Epidemiology División, UMAE HES, Western National Medical Center, Guadalajara 44340, Mexico; erksland@gmail.com

**Keywords:** dengue virus, DENV, arbovirus, outbreak, epidemiology, prevention, Mexico

## Abstract

Dengue is considered the most prevalent mosquito-borne arboviral disease worldwide, representing a public health challenge as its incidence has tripled in the last 30 years. The World Health Organization reports 390 million infections annually in more than 129 countries, with approximately 96 million symptomatic cases and around 40,000 deaths. Mexico is a hyperendemic country, with high prevalence and significant outbreaks. In 2024, a surge was observed, with approximately 125,000 infections and nearly 480 deaths. The states with the most cases and deaths were Colima and Jalisco, respectively, placing significant strain on healthcare services and driving up costs. The disease’s epidemiology from 2014 to 2025 is characterized by marked seasonality and periodicity, and by the simultaneous circulation of all four serotypes. In recent years, a notable increase in DENV-3 has been observed. In 2025, there were 21,981 confirmed cases; Sonora recorded the highest incidence, while Jalisco and Sinaloa reported the highest number of deaths. This study provides a unique decadal analysis of the epidemiological characteristics of dengue in Mexico, highlighting potential challenges and emphasizing the importance of epidemiological surveillance and future approaches, such as vaccine provision in the country, to mitigate the high mortality rate and associated costs.

## 1. Introduction

Vector-borne diseases cause approximately 700,000 deaths annually, and more than 80% of the global population is at risk of infection, with mosquito-borne diseases representing the most prevalent group. Therefore, mosquito vector control is currently the most effective measure within global public health strategies. However, it is a highly complex task due to climatic, geographic, demographic, sociocultural, and urbanization factors, as well as international travel, the emergence of insecticide resistance, and other challenges. Altogether, these factors underscore the need for reliable preventive indicators to improve disease control, along with continuous evaluation of the epidemiological situation and planning to prioritize resource allocation in public health [1,2].

Dengue is a major threat to humanity, as it is one of the most rapidly spreading vector-borne viral diseases and has one of the longest histories of being a public health concern [3]. In the last 30 years, the incidence of dengue has increased threefold [4,5]. According to the World Health Organization (WHO), DENV infection affects approximately 390 million people each year in more than 129 countries, with an estimated 96 million symptomatic cases, around 40,000 annual deaths [1,6], and a global case fatality rate of 0.11% (in 2023), increasing to 0.43% in Africa [7]. Furthermore, about half of the world’s population lives in dengue-endemic or hyperendemic areas [8,9]. Globally, approximately 5 million cases were recorded in 2023, rising to 14 million in 2024 [10,11]. Low-income settings, pollution, climate change, immigration, and other factors [12] have created an ideal environment for mosquito development and spread, leading to the emergence of dengue in non-endemic regions and to case numbers exceeding previous expectations [13].

Humans are considered the main host, carrier, and amplifier of the virus, along with non-human primates, which also fulfill this role in the sylvatic cycle [2,14,15]. Dengue is maintained through two main mechanisms: horizontal transmission of the virus between mosquitoes and humans and vertical transmission from infected females to their offspring [16]. Consequently, modeling studies estimate that by 2080, around 63% of the global population will reside in areas at risk of dengue transmission [17].

Dengue is an acute disease caused by the dengue virus (DENV), an arbovirus comprising four serotypes (DENV-1-DENV-4), which is transmitted to humans through infected mosquitoes of the genus Aedes, mainly *Aedes aegypti* and, to a lesser extent, *Aedes albopictus* [2,7,18,19].

The different serotypes of dengue are distributed worldwide in tropical and subtropical endemic areas (where a single serotype predominates) and hyperendemic areas (where multiple serotypes co-circulate within the same region), as is the case in Mexico [18,20]. These serotypes are closely related genetically but antigenically distinct [2]. Given its geographical location and ecological characteristics, Mexico remains a hyperendemic country within the Americas, experiencing recurrent outbreaks, as well as the co-circulation of multiple viral serotypes [16]. In this context, critical points or persistent transmission zones are specific geographical areas where transmission is concentrated [16].

In hyperendemic settings where multiple dengue virus serotypes co-circulate, immune interactions between sequential infections play a critical role in disease severity. Antibody-dependent enhancement (ADE) occurs when pre-existing, non-neutralizing antibodies facilitate viral entry into Fcγ receptor-bearing cells, promoting increased viral replication and immunopathology [21,22].

This situation not only impacts the morbidity and mortality of the population but also places considerable pressure on health systems and generates substantial economic losses associated with medical care and labor productivity [23,24].

The objective of this narrative review is to offer a comprehensive and up-to-date overview of dengue in Mexico over the last decade, encompassing crucial aspects such as the clinical characteristics of the disease, its epidemiology and spatio-temporal distribution, current clinical management, advances in vaccines, the costs associated with the disease, and future prospects for developing control strategies within the country.

## 2. Materials and Methods

A retrospective descriptive analysis was conducted on epidemiological data from Mexico from 2014 to 2025. Data were sourced from the National System for Epidemiological Surveillance (SINAVE/DGE/SALUD), specifically the Dengue Epidemiological Surveillance System. This public health surveillance infrastructure operates in accordance with the Official Mexican Standard NOM-017-SSA2-2012 [25] and the Manuals for Epidemiological Surveillance of Vector-Borne Diseases. 

For the data analysis, federal-level information compiled by this agency from all Mexican states was aggregated. The dataset comprised confirmed cases analyzed both on an annual basis and by epidemiological week. Data were analyzed with Prism software v9.1.1 (GraphPad Software).

A chronological analysis of the annual data was performed to determine case counts, incidence rates, mortality, clinical classification, and the distribution of serotypes at the federal level. Furthermore, an in-depth analysis of the year 2025 was conducted by epidemiological week, examining cases, incidence, clinical classification, and prevalent serotypes categorized by federal entity.

## 3. Epidemiological Features of Dengue Outbreaks in Mexico

In Mexico, according to a 2014 article, between 1990 and 2011, the number of dengue cases did not increase substantially, but the disease became more severe. Specifically, 20% of cases were dengue hemorrhagic fever (DHF) from 2002 onwards, and outbreaks tended to last longer. Furthermore, during the latter decade studied, cases increased in the south of the country, and increased dengue cases among adolescents and children, and increased DHF incidence were recorded [18].

**Figure 1:**
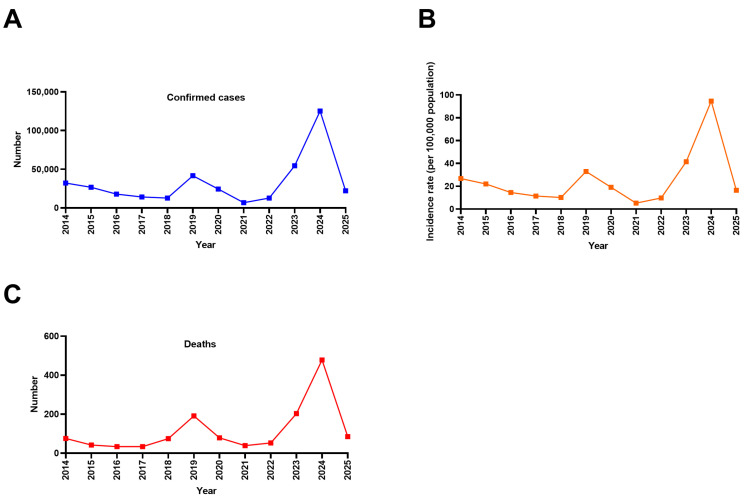
Dengue cases, deaths, and incidence in Mexico, 2014–2025. (**A**) Confirmed cases, (**B**) incidence rates per 100,000 population, and (**C**) dengue-related deaths. Figure generated by the authors based on data from SINAVE/DGE/SALUD/Special Epidemiological Surveillance System for Dengue (accessed on 8 January 2026).

To better understand recent dynamics, here, we analyze epidemiological data on dengue in Mexico for 2014–2025. Figure 1A,B illustrate the epidemiological peaks that occurred in 2014, 2019, and 2024. Each peak lasted approximately three years, followed by periods of low incidence. Figure 1B shows that the years with the highest incidence were 2019 and 2024, with approximately 30 and 100 cases per 100,000 inhabitants, respectively. A similar epidemiological pattern was observed for dengue-related deaths. Thus, in the last decade, the year with the highest number of cases and the highest mortality was 2024, with approximately 150,000 total cases and 500 deaths recorded in the same year (Figure 1A,C).

Regarding epidemiological dynamics, several concepts must be considered, such as the impact on the magnitude of an epidemic, including the proportion of symptomatic and asymptomatic infections, which is influenced by immune responses such as neutralizing antibodies generated by previous infections. In this regard, the initial and subsequent infecting serotypes, as well as the genotype, clade, or strain, are important determinants of prognosis, influencing whether infection is asymptomatic or symptomatic, as well as disease severity at both the individual and population levels [2].

During the last decade, there were two surges in dengue cases worldwide. The first occurred in 2019. In the two subsequent years, a decreased number of cases was observed, suggesting that this decline may have been partially attributable to reduced detection and reporting during the COVID-19 pandemic, rather than exclusively to changes in viral transmission. In 2022, global cases began to increase again [26], and by 2024, a second historic surge was documented, with 14.1 million cases reported worldwide. This figure not only exceeds the 7 million cases registered in 2023 but also doubles them and represents a twelve-fold increase compared with the 1,206,644 cases reported in 2014. Despite the high infection rate, the disease maintained an overall case fatality rate of 0.07%, with 9508 related deaths [11,27].

In an article compiling dengue cases in Mexico from 2022 to 2024 across 2471 localities nationwide, it is reported that cases increased during this period by 38% to 68.6%. A predominance of the DENV-2 serotype was observed in 2022, followed by a transition to DENV-3 in 2023 and 2024, together with a geographical shift toward the Pacific coast and southern areas and from central regions toward the southeast [16].

**Figure 2:**
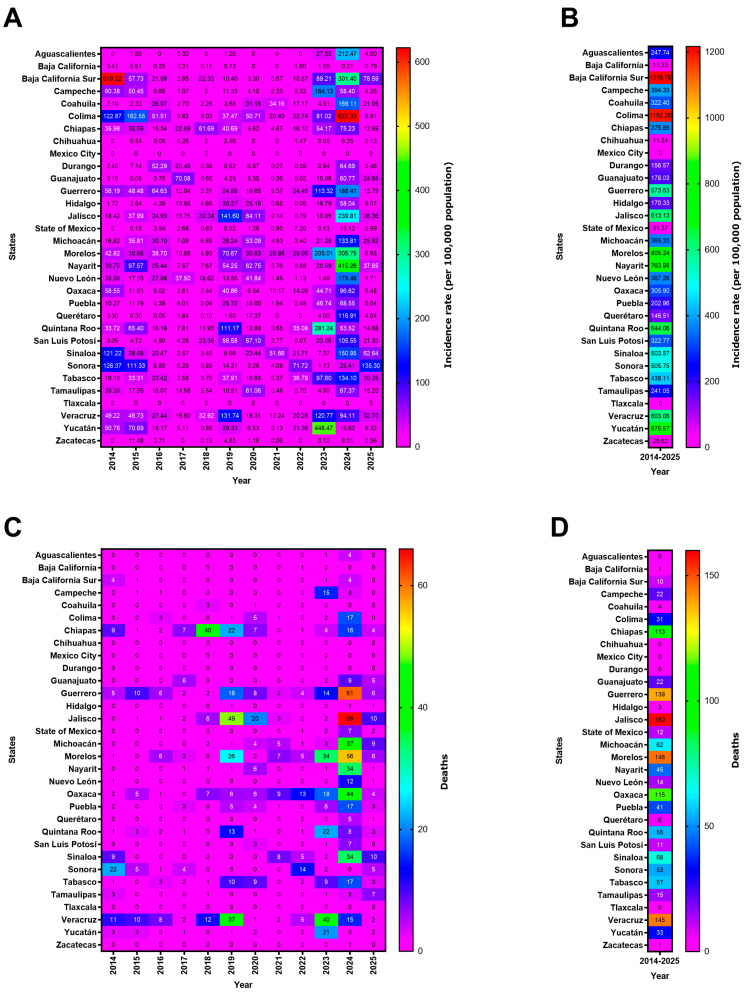
Incidence rates for and deaths from dengue in the states of Mexico, 2014–2025. (**A**) Incidence rates per 100,000 people, (**B**) cumulative incidence rates per 100,000 people (2014–2025), (**C**) dengue-related deaths, and (**D**) cumulative dengue-related deaths. Figure generated by the authors based on data from SINAVE/DGE/SALUD/Special Epidemiological Surveillance System for Dengue (accessed on 8 January 2026).

Based on the information analyzed in this study, Figure 2 shows the incidence of dengue cases per 100,000 inhabitants reported in the different states of Mexico over the last decade. In 2014, the states with the highest incidence rates were Baja California Sur (*n* = 618.32), Sonora (*n* = 126.37), and Colima (*n* = 122.87). In 2019, the three states with the highest number of cases were Jalisco (*n* = 141.60), Veracruz (*n* = 131.74), and Quintana Roo (*n* = 111.17). During the epidemiological peak of 2024, the reported data stated that the states with the highest prevalence rates were Colima (*n* = 622.33), Nayarit (*n* = 415.26), and Morelos (*n* = 305.75). Mexico City and Tlaxcala were the only states with no reported dengue cases throughout the entire decade (Figure 2A).

Figure 2B presents an analysis of cumulative incidence, showing that the states with the highest incidence rates, ranked by number of cases, were Baja California Sur (*n* = 1216.76), Colima (*n* = 1192.26), Morelos (*n* = 805.24), Nayarit (*n* = 763.98), Yucatán (*n* = 675.57), Quintana Roo (*n* = 644.06), Jalisco (*n* = 613.13), Veracruz (*n* = 603.06), and Guerrero (*n* = 573.53). In this same analysis, the states with the lowest cumulative incidence rates over the last 10 years were Chihuahua (*n* = 11.24), Baja California (*n* = 11.33), Zacatecas (*n* = 29.62), and the State of Mexico (*n* = 31.37).

Using the same approach for dengue-related deaths, Figure 2C shows that in 2014, the states with the highest number of deaths were Sonora (*n* = 22), Veracruz (*n* = 11), and Sinaloa (*n* = 9). In 2019, they were Jalisco (*n* = 49), Veracruz (*n* = 37), and Morelos (*n* = 26). At its peak in 2024, the data for the states with the highest incidence rates were as follows: Jalisco (*n* = 66), Guerrero (*n* = 61), and Morelos (*n* = 56).

The analysis of cumulative deaths from 2014 to 2025 (Figure 2D) indicates that the states with the highest number of deaths were Jalisco (*n* = 160), Morelos (*n* = 146), Veracruz (*n* = 145), Guerrero (*n* = 139), Oaxaca (*n* = 115), and Chiapas (*n* = 113). The states with the lowest number of deaths were Chihuahua (*n* = 0), Durango (*n* = 0), Mexico City (*n* = 0), Tlaxcala (*n* = 0), Zacatecas (*n* = 1), Baja California (*n* = 1), Hidalgo (*n* = 3), and Coahuila (*n* = 4).

## 4. Clinical Characteristics and Spatio-Temporal Distribution of Dengue Virus Serotypes in Mexico

A primary dengue infection occurs when a person is infected with a dengue virus serotype for the first time. Following this initial infection, the individual develops lifelong immunity to that specific serotype but only short-lived cross-immunity to the other three serotypes. Reinfection with a different serotype is termed a secondary, or heterotypic, infection and increases the risk of severe disease. Notably, this increased risk does not appear with third or fourth infections [7]. Reinfection by a different serotype is considered the greatest risk factor for severe clinical manifestations due to ADE [22,28,29].

The clinical spectrum of dengue ranges from asymptomatic infections to severe multi-organ involvement (heart, liver, kidneys, and brain, among others) that may require intensive care. Typical symptoms resemble those of influenza, including sudden-onset high fever (up to 40 °C); frontal headache; retro-orbital pain; muscle and joint pain; back pain; nausea and vomiting; palpitations; rash; and, in severe cases, hemorrhage or circulatory shock. These signs should prompt suspicion of dengue in patients residing in endemic tropical or subtropical areas, or in travelers from these regions [6,30,31].

Currently, dengue can be diagnosed by detecting viral RNA or by serological testing for the NS1 antigen during the first 5 days of illness. For infections lasting longer than five days, the detection of specific IgM and IgG antibodies is recommended [32,33]. Accurate diagnosis is closely linked to dengue classification, which has evolved internationally to improve timely detection and clinical management.

Clinical management of dengue in Mexico is currently governed by the most recent algorithms published in January 2025 (1st edition) by the Ministry of Health’s National Center for Disease Control and Prevention (CENAPRECE), which adhere to the standardized protocols established by the PAHO and the WHO. These guidelines constitute the regulatory and clinical framework for the management of dengue patients within the Mexican healthcare system [34].

The WHO initially categorizes dengue into two main groups: dengue fever (DF) and dengue shock syndrome (DSS). Symptomatic cases range from mild DF to potentially fatal DHF, with children generally experiencing milder symptoms than adults [35]. Severe cases, such as DHF, are characterized by plasma leakage into pleuroperitoneal spaces, requiring careful monitoring and fluid replacement [31].

Despite its utility, the original classification had important limitations, as it failed to capture all severe cases. To address this, the WHO revised its guidelines in 2009, emphasizing that disease severity is primarily determined via plasma leakage rather than bleeding. The updated system also facilitated epidemiological surveillance at the primary care level, without requiring sophisticated laboratory tests [17,36,37,38]. Prior to this classification, approximately 32% of severe cases were not recognized [39]. The new classification established a three-group system based on clinical management: dengue without warning signs (DWWSs), dengue with warning signs (DWSs), and SD [40].

These guidelines were adopted by the Pan American Health Organization (PAHO) the same year for use in the Americas. Most countries incorporated them into national protocols within five years, thereby improving early detection, clinical management, and care of severe cases without relying on the laboratory-based criteria of the previous classification [41]. It is important to note that during 2014–2015, Mexico was transitioning its surveillance system. Consequently, epidemiological reports from that time reflected the older DF/DHF classification, even as the updated framework was being implemented. Since 2016, has adopted a surveillance framework classifying dengue cases into four distinct groups: DWWS, DWS, SD, and DWS + SD. This system allows for detailed monitoring of disease progression and severity within the Mexican national health system.

**Figure 3:**
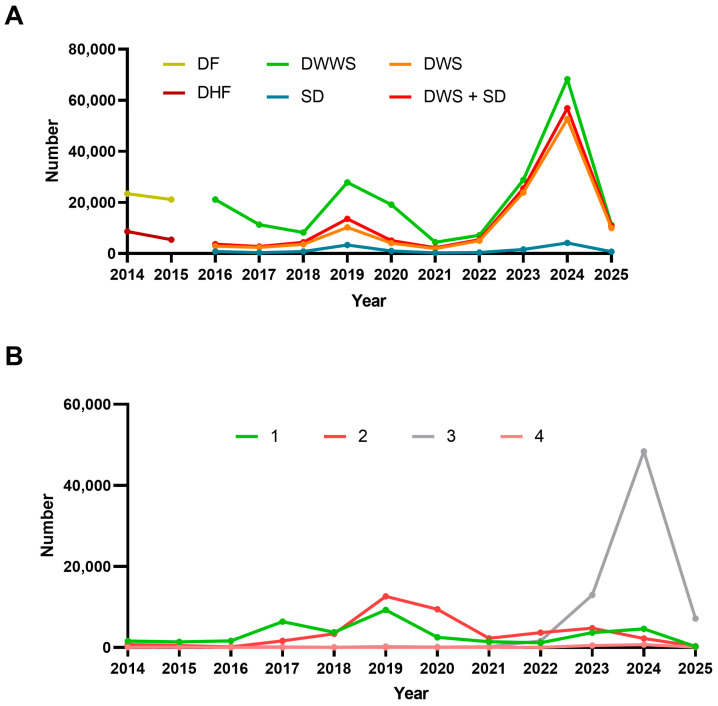
Clinical classification and serotype distribution of dengue cases in Mexico, 2014–2025. (**A**) Clinical classification: dengue fever (DF) and dengue hemorrhagic fever (DHF) were reported in 2014–2015. From 2015 onwards, cases were classified as dengue without warning signs (DWWSs), dengue with warning signs (DWSs), and severe dengue (SD) and DWS + SD. (**B**) Distribution of DENV serotypes (DENV-1 to DENV-4). Figure generated by the authors based on data from SINAVE/DGE/SALUD/Special Epidemiological Surveillance System for Dengue (accessed on 8 January 2026).

Figure 3 shows the incidence of the clinical forms and circulating serotypes in Mexico over the last 10 years. Regarding the clinical forms (Figure 3A), prior to 2015, under the two-group classification (DF and DHF), most cases were DF. From 2016 onwards, with the adoption of the four-group classification, epidemic peaks were observed in 2019 and 2024 across all clinical forms, with approximately 10,000 and 60,000 cases of DWS + SD, respectively. These trends mirror the observed patterns for deaths. Figure 3B depicts the distribution of serotypes over a 10-year period. Serotype 1 predominated until 2018, while serotype 2 emerged and dominated between 2019 and 2022. In 2023, serotype 3 became predominant, affecting a population with little to no pre-existing immunity. In 2024, PAHO issued an epidemiological alert for the resurgence of DENV-3, which had been absent in certain areas for several years [42]. Serotype 4 remained the least prevalent serotype throughout the decade, highlighting the need for ongoing epidemiological surveillance.

Based on these trends, it is reasonable to expect, with some reservations, that fewer dengue cases may occur in the next two years, with a potential new peak around 2028–2029.

**Figure 4:**
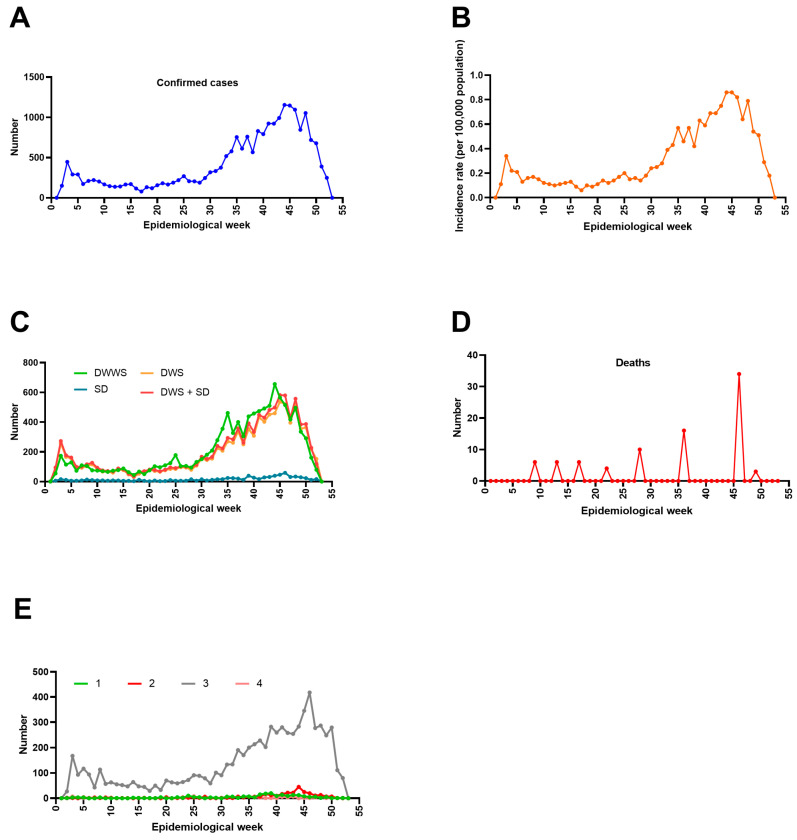
Current epidemiological situation for dengue in Mexico, 2025. (**A**) Confirmed cases. (**B**) Incidence rates per 100,000 people. (**C**) Clinical classification: dengue fever (DF) and dengue hemorrhagic fever (DHF) were reported in 2014–2015. From 2015 onwards, cases were classified as dengue without warning signs (DWWSs), dengue with warning signs (DWSs), and severe dengue (SD) and DWS + SD. (**D**) Dengue-related deaths and (**E**) the distribution of DENV serotypes (DENV-1 to DENV-4). An epidemiological week begins on Sunday and ends on Saturday. The year 2025 is divided into 53 epidemiological weeks based on this weekly cycle. Figure generated by the authors based on data from SINAVE/DGE/SALUD/Special Epidemiological Surveillance System for Dengue (accessed on 8 January 2026).

Regarding the epidemiological situation for dengue in Mexico in 2025, Figure 4A highlights a small increase in cases during the first five epidemiological weeks, followed by a more pronounced increase from week 30, coinciding with the rainy season. A similar pattern is observed for the incidence per 100,000 inhabitants (Figure 4B). The most prevalent clinical forms, DWWS, DWS, and DWS + SD, also exhibit parallel trends, with marked increases starting in week 30 (Figure 4C). The highest number of dengue-related deaths is observed in week 46 (Figure 4D). Regarding serotype distribution, DENV-3 was the most prevalent serotype throughout 2025, while DENV-1 and DENV-2 were observed less frequently, and DENV-4 was almost entirely absent (Figure 4E).

## 5. Seasonal Occurrence of Dengue Outbreaks in Mexico

Despite vector control efforts, mosquito populations and mosquito-borne disease prevalence continue to increase globally, and dengue continues to exhibit cyclical outbreak patterns [1,18]. Consequently, epidemic outbreaks occur worldwide every 3–5 years [43].

Seasonality is a major factor influencing the population dynamics of *Aedes aegypti*. In a study carried out by Pliego E. and collaborators in 2017, a dynamic model was used to evaluate the effects of temperature and precipitation on mosquito abundance. The authors also analyzed the correlation between mosquito populations and dengue outbreaks using historical data from eight Mexican regions, as well as the role of diapause in seasonal outbreaks.

This study revealed that mosquito abundance is governed by the synchrony of multiple ecological mechanisms, which can either enhance or inhibit population growth. Notably, the findings demonstrate that seasonal minimum temperatures, rather than mean temperature values, act as the primary biological driver for mosquito population peaks. These thermal lows function as a critical threshold that regulates the termination of diapause and the subsequent timing of outbreaks; thus, the abatement of seasonal cold, when synchronized with rainfall, triggers the rapid expansion of the vector population and the consequent escalation of dengue transmission [44].

## 6. Challenges Associated with the Increasing Number of Dengue Cases in Mexico—Epidemiological Situation in 2025

The challenges facing the health system due to the increasing number of dengue cases in Mexico are highly complex, as multiple factors contribute to this increase.

Among the reported drivers, increases in air temperature, sea surface temperature (SST), rainfall, and El Niño–Southern Oscillation (ENSO) have been associated with higher case numbers in certain regions [45]. Dengue transmission in the warm and humid regions of Mexico has been shown to be strongly influenced by ENSO and other climatic variables [46,47].

Consequently, climate change, combined with increased global trade and tourism, has been identified as a major factor contributing to the growing incidence of dengue. Furthermore, this is compounded by the mosquito’s adaptation to higher altitudes, and Mexico offers ideal conditions for dengue due to its extensive coastal and tropical areas, population density, and economy primarily based on foreign trade and tourism [43]. Moreover, continuous mosquito breeding, with peak reproductive activity typically occurring at the onset of the rainy season [43], further contributes to the seasonal increase in dengue cases. In Mexico, which has a monsoon climate, the rainy season spans the summer months (June to September), followed by a relatively dry winter [48]. According to our data analysis, this period, corresponding to epidemiological weeks 22–39, coincides with the upward inflection point, or accelerated growth phase, in dengue cases and incidence (Figure 4A,B).

**Figure 5:**
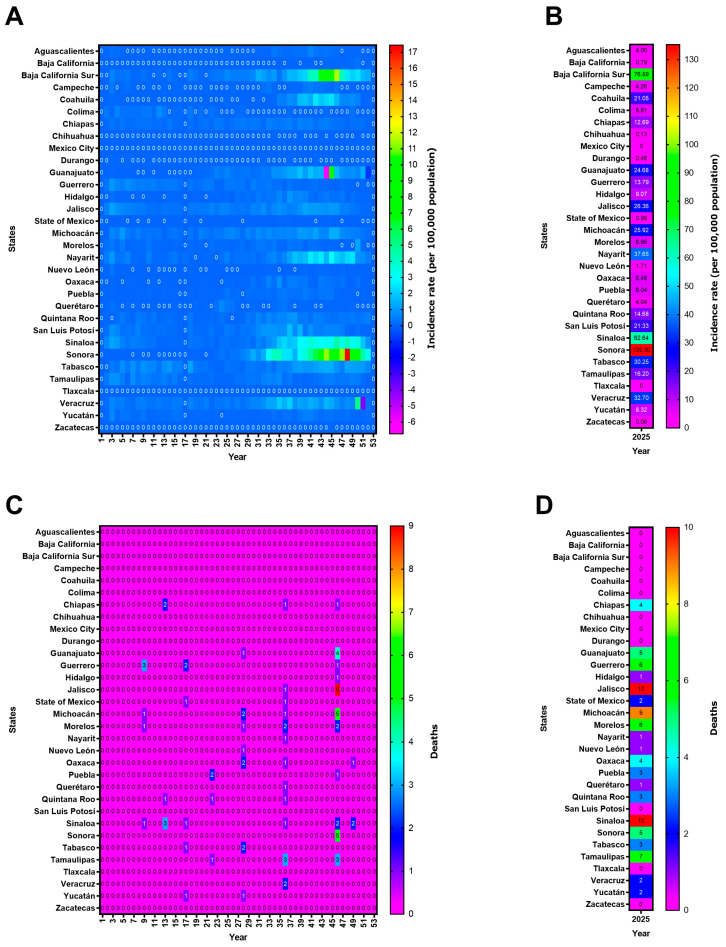
Incidence rates and dengue-related deaths in the states in Mexico, 2025. (**A**) Incidence rates per 100,000 people, (**B**) cumulative incidence rates per 100,000 people (2014–2025), (**C**) dengue-related deaths, and (**D**) cumulative dengue-related deaths. An epidemiological week begins on Sunday and ends on Saturday. The year 2025 is divided into 53 epidemiological weeks based on this weekly cycle. Figure generated by the authors based on data from SINAVE/DGE/SALUD/Special Epidemiological Surveillance System for Dengue (accessed on 8 January 2026).

To examine the situation in more detail, incidence rates and dengue-related deaths across Mexican states in 2025 were analyzed (Figure 5). The results showed that Sonora, Baja California Sur, and Sinaloa experienced the earliest increases in incidence, starting at around epidemiological weeks 25–30 (Figure 5A). The highest cumulative incidence per 100,000 inhabitants corresponded to the states of Sonora (*n* = 135.3), Baja California Sur (*n* = 76.59), Sinaloa (*n* = 62.64), Nayarit (*n* = 37.65), Veracruz (*n* = 32.7), Tabasco (*n* = 30.25), and Jalisco (*n* = 26.36) (Figure 5B). The first dengue-related deaths in 2025 occurred in epidemiological week 9, primarily in Guerrero (*n* = 3), Michoacán (*n* = 1), Morelos (*n* = 1), and Sinaloa (*n* = 1) (Figure 5C). When the cumulative deaths were analyzed by state, the most represented states were Jalisco (*n* = 10), Sinaloa (*n* = 10), Michoacán (*n* = 9), Tamaulipas (*n* = 7), Guerrero (*n* = 6), Morelos (*n* = 6), Guanajuato (*n* = 5), Sonora (*n* = 5), and Chiapas (*n* = 4) (Figure 5D).

## 7. Vaccines

Currently, no specific drug or universally effective vaccine is available for mosquito-borne diseases such as dengue [1]. Therefore, several dengue vaccines have been developed to combat the disease, though challenges remain and improvements are still needed.

CYD-TDV (Dengvaxia^®^, Sanofi Pasteur (Lyon, France)) was the first vaccine authorized in some countries, and it was introduced in Brazil in 2015 [49,50]. It is a live-attenuated tetravalent yellow fever virus-derived vaccine with a three-dose regimen administered at 6-month intervals and is generally recommended for individuals aged 9 to 45 years [7,49]. The Advisory Committee on Immunization Practices (ACIP) recommends it for children aged 9 to 16 years living in endemic areas with laboratory-confirmed DENV infection [49]. It is also recommended when seroprevalence in this age group exceeds 80% [51]. The WHO initially recommended its use in populations with seroprevalence greater than 70%, but since 2018, vaccination strategies have been limited to seropositive individuals [50]. The main limitations of this vaccine include the requirement for prior serological testing to determine effectiveness, its lower efficacy against the DENV-2 serotype, its reduced effectiveness in children under 9 years of age or without prior exposure to the virus, and the increased risk of hospitalization and severe dengue in vaccinated children aged 2 to 5 years [18,49]. Despite these limitations, the vaccine induces strong seroconversion and neutralizing antibody responses against all four serotypes. In preclinical studies conducted in monkeys, it conferred 92% protection [52]. In a Latin American clinical trial with 20,869 healthy children aged 9–16 years, overall efficacy was 60.8% after 13 months, with 80.3% efficacy in preventing hospitalization due to severe dengue, highlighting its potential to reduce healthcare burden [7].

The TAK-003 or Qdenga vaccine (Takeda Vaccines (Tokyo, Japan), Cambridge, MA, USA) is a two-dose vaccine administered three months apart and derived from DENV-2. It has been approved for use in the European Union, the United Kingdom, and several endemic countries (Brazil, Thailand, Indonesia, Argentina) [7,8,49]. In a study conducted by Tricou, V. et al. [8], this vaccine showed an overall efficacy of 80.2% against biologically confirmed dengue. In seropositive populations, the vaccine was effective against all four serotypes. In seronegative populations, efficacy was 45.4% for DENV-1 and 88.1% for DENV-2, with limited data for DENV-3 and DENV-4 due to low prevalence. The vaccine also reduced severe cases and clinical complications, with 95.4% efficacy in preventing hospitalization 12 months after the last dose [7,8]. Overall, this vaccine has demonstrated an overall robust efficacy of 73.3% against symptomatic dengue and 90.4% against hospitalization, regardless of serological status. Its protection against the DENV-2 serotype is particularly noteworthy, with a high efficacy of 97.7% reported in phase III trials [53].

TV003/TV005 (manufactured by the U.S. National Institute of Allergy and Infectious Diseases, Bethesda, MD, USA) is another vaccine candidate constructed with live-attenuated viruses. It is a single-dose vaccine in phase III development that has shown efficacy in rhesus macaques against the wild-type DENV-1 and DENV-4 serotypes [49,52,54,55]. A single-dose administration of the TV003 and TV005 vaccines showed high immunogenicity. The TV003 formulation achieved tetravalent seroconversion against all four DENV serotypes in 74–92% of seronegative adults, with TV005 showing a similar rate of 90% [56].

Another vaccine that has recently completed Phase III development is Butantan-DV, a tetravalent analog developed using the U.S. National Institutes of Health (NIH)’s TV003 technology in collaboration with the Butantan Institute and the American Type Culture Collection (ATCC). This live-attenuated virus vaccine received regulatory approval from Brazil’s National Health Surveillance Agency (ANVISA) in November 2025 [57]. Clinical studies reported an overall efficacy of 79.6% against any serotype at two years, specifically showing protection against DENV-1 and DENV-2 regardless of prior serological status. Notably, efficacy in previously infected patients reached 89.2% [7,49]. Different Phase 1, 2, and 3 studies have demonstrated acceptable side effects regardless of prior exposure; thus, Butantan-DV stands to become the first single-dose quadrivalent dengue vaccine to be approved for widespread use [49].

Challenges in dengue vaccine development include the lack of optimal animal models for testing, the need for vaccines effective across all serotypes, and the risk of ADE, in which exposure to one serotype can produce sub-neutralizing responses to others [28,49,52]. Additionally, limited awareness of vaccine guidelines among healthcare personnel, even in endemic regions, can reduce coverage and lead to suboptimal use [7].

In Mexico, the TAK-003 dengue vaccine is currently undergoing the standard regulatory review process for potential inclusion in public health programs. Its successful adoption depends on overcoming challenges with clinical implementation, including strict adherence to guidelines and sufficient training of healthcare staff. The prospective implementation of serostatus-independent vaccines with broadened age indications offers a strategic opportunity to streamline clinical decision-making. Current examples include TAK-003 (Qdenga^®^), a tetravalent, two-dose regimen administered over a three-month interval [8], and the single-dose Butantan-DV vaccine. Furthermore, the transition to reduced dosing schedules mitigates the logistical complexities of multi-stage booster administrations in three- or six-month increments; such refinements are paramount for effective public health campaigns in regions such as Mexico and Brazil [49].

## 8. Clinical Management of Dengue

Dengue can be symptomatic or asymptomatic. In symptomatic cases, the disease typically progresses through three stages: febrile, critical, and recovery [58]. Furthermore, depending on the characteristics of the clinical manifestation, cases can be organized into three specific groups regarding clinical management: Group A—treatable at home; Group B—requires hospital treatment; Group C—requires emergency treatment [59].

Group A comprises patients who can be treated at home, due to specific characteristics such as tolerating adequate volumes of oral fluids and urinating at least once every six hours, without presenting any alarming signs. Management recommendations include rest and adequate fluid intake, as this can prevent hospitalization [60]. However, it is important to emphasize that daily auscultation is essential for assessing progression [61].

Group B includes dengue patients who require hospitalization for observation and management during the critical phase, especially those with warning signs (such as severe abdominal pain, persistent vomiting, mucosal bleeding, drowsiness, hepatomegaly, or fluid accumulation) or associated conditions that complicate the disease, such as pregnancy, lactation, childhood, old age, obesity, diabetes mellitus, renal failure, or hemoglobinopathies, as well as patients with social limitations that prevent adequate follow-up [61,62]. Treatment is based on the controlled administration of isotonic crystalloid solutions (0.9% sodium chloride, or Ringer’s lactate), starting with 5–7 mL/kg/h for 1–2 h, gradually reducing to 3–5 mL/kg/h for 2–4 h and then to 2–3 mL/kg/h, adjusting the dosage according to clinical evolution and hematocrit [58,63]. Intravenous fluids are administered only with the minimum volume necessary to maintain good perfusion and a diuresis of at least 0.5 mL/kg/h, and should be gradually reduced as capillary leakage decreases [64]. Patients should be closely monitored, with vital signs, peripheral perfusion, urine output, hematocrit, and renal and hepatic function checked every few hours until the critical period has passed [65]. In patients with no alarm symptoms, oral hydration is encouraged, with intravenous fluids initiated only if oral fluids are not tolerated, with frequent adjustments and constant monitoring of clinical and laboratory status [61,62].

Group C comprises patients with severe dengue who require emergency treatment and immediate referral to a hospital with critical care capacity. These cases are characterized by severe plasma leakage, which can lead to shock or respiratory distress, severe bleeding, or damage to major organs (such as the liver, kidney, and heart), as well as to neurological disorders [61,64]. Management consists of immediate fluid resuscitation with isotonic crystalloid solutions (e.g., 0.9% saline or Ringer’s lactate) at 5–10 mL/kg/h for the first hour, adjusting the rate according to the clinical response and hematocrit [58,63]. If shock persists, 10–20 mL/kg crystalloid or colloid boluses are administered under close monitoring, and the need for blood transfusion is assessed in cases of hemorrhage [58,65]. The goal of treatment is to restore perfusion and hemodynamic stability while avoiding fluid overload, and thus, maintaining a urine output ≥ 0.5 mL/kg/h [62].

In summary, effective management of dengue depends on accurate stratification according to disease severity. Group B patients should be hospitalized to prevent complications, while Group C patients require immediate fluid resuscitation, intensive monitoring, and, in selected cases, blood transfusion to restore tissue perfusion without causing fluid overload [58,61]. Hydration guided by clinical and laboratory parameters, such as hematocrit, is the most effective therapeutic approach for reducing mortality and the complications of severe dengue [62,64]. Furthermore, the correct application of these clinical guidelines, which prioritize the use of isotonic crystalloid solutions and close monitoring during the critical phase, has been shown to significantly improve clinical outcomes and reaffirm the importance of training healthcare personnel and implementing standardized protocols at all levels of care [58,63,65].

Mexico’s national health institutions have formally adopted and reinforced these WHO/PAHO guidelines, ensuring standardized, evidence-based management focused on early stratification and timely intervention.

## 9. The Costs of Dengue

Mosquito-borne diseases, including dengue, impose a substantial economic burden worldwide. In 2013, the estimated global cost of dengue reached approximately USD 8.9 billion [1]. The economic impact of dengue extends far beyond hospitalization expenses, encompassing lost productivity, decreased economic activity, and broader societal costs in both fatal and non-fatal cases [7,17].

In Mexico, Zubieta-Zavala et al. reported that in 2012, the average treatment costs for DF were USD 32.60 for outpatient care and USD 490.93 for inpatient care according to the Secretariat of Health (SS), as well as USD 92.03 for outpatient care and USD 1644.69 for inpatient care according to the Mexican Social Security Institute (IMSS). In both cases, these figures fell below the estimated ideal treatment costs, highlighting deficiencies in patient care for DF [66].

Latin America is one of the regions with the greatest economic burden attributable to dengue, representing approximately 38% of the estimated global cost, due to the high incidence and number of annual hospitalizations. These costs are amplified by absenteeism from work, household expenses for treatment and transportation, and losses in the tourism sector during outbreaks [24,67,68].

Furthermore, recent studies emphasize that the economic impact of dengue is not confined to hospital care. In endemic regions of Mexico and Central America, dengue outbreaks significantly reduce household income due to lost workdays and out-of-pocket medical expenses [17]. The combination of direct healthcare costs and indirect socio-economic losses makes dengue one of the most financially burdensome vector-borne diseases in middle-income countries, where health systems often operate under budget constraints [63].

Therefore, investment in sustainable vector control, vaccination, and health education programs not only reduces dengue-related morbidity and mortality but also constitutes a cost-effective, long-term strategy for health systems. Economic modeling suggests that effective prevention and early detection measures could potentially reduce total dengue-related costs in Latin America by up to 40% [24,63,68].

## 10. Future Perspectives on Dengue Outbreaks in Mexico

In recent years, Mexico has experienced recurrent dengue outbreaks in which all four serotypes of the virus (DENV-1, DENV-2, DENV-3, and DENV-4) have circulated. Historically, DENV-1 and DENV-2 have predominated in most cases, while DENV-4 has had limited circulation, representing a very small proportion of infections nationwide [69]. However, as demonstrated in this study, DENV-3 has recently become markedly predominant relative to the other serotypes, particularly DENV-4.

This low prior exposure implies that a large proportion of the Mexican population lacks immunity to the DENV-4 serotype, creating favorable conditions for a future outbreak of significant magnitude should this serotype become predominant. Similar phenomena have been observed in other Latin American countries, where the reemergence of a rare serotype has triggered widespread transmission and an increased disease burden [70].

From an immunological perspective, infection with one dengue serotype confers long-term immunity only to that specific serotype, without protection against others. Moreover, secondary infections with a different serotype are associated with an increased risk of severe disease due to the ADE phenomenon [71]. Therefore, if DENV-4 becomes predominant in the future, the proportion of severe cases could increase, as many individuals may experience secondary infection with a heterologous serotype [72,73]. Seroprevalence studies in endemic regions such as Morelos and Veracruz have revealed heterogeneous levels of neutralizing antibodies, confirming low population immunity against DENV-4 [73].

Environmental and climatic factors further influence the potential reemergence of DENV-4. Predictive models of spatial distribution indicate that large tropical and subtropical regions of Mexico, particularly the coastal areas of the Pacific and the Gulf, present high environmental suitability for the transmission of this serotype, with suitability reaching up to 85% in some municipalities due to temperature, humidity, and vector density (Aedes aegypti) [74,75]. Contributing factors such as rapid urbanization, increased human mobility, and insufficient vector control further increase the risk of the introduction and dissemination of DENV-4. Consequently, coastal and peri-urban areas are expected to be most vulnerable to a potential large-scale outbreak dominated by this serotype [76].

In anticipation of this scenario, it is a priority to strengthen genomic, serotype-specific, and entomological surveillance, as well as vector prevention and control strategies. Introducing quadrivalent vaccines that offer protection against DENV-4 represents a promising tool for reducing disease incidence in regions with low immunity [61,64]. Similarly, healthcare personnel training, community communication, and timely care in medical services are key to containing potential outbreaks. Finally, using scientific evidence and regional risk models to anticipate epidemiological threats will enable the development of more effective public health policies in the event of DENV-4 gaining predominance in Mexico [58,64].

## 11. Discussion

Given the current epidemiological landscape in Mexico, which is characterized by a significant increase in severe DENV cases, securing sustained and dedicated public funding has become critical to prevent the saturation and potential collapse of national health systems. This urgency is reflected in the most recent surveillance data from 2025, in which Sonora reported the highest cumulative incidence nationwide, while Jalisco and Sinaloa exhibited the highest dengue-associated mortality rates, underscoring pronounced regional heterogeneity in disease burden.

A comprehensive analysis of the last decade confirms that dengue in Mexico and Latin America remains a complex and continuously evolving public health challenge, posing an increasing threat to population health. Although substantial progress has been achieved in understanding clinical manifestations and standardizing case management, dengue epidemiology continues to demand constant surveillance and adaptive response strategies. The progressive increase in outbreak frequency and spatial expansion observed in recent years aligns with reports from the PAHO and the WHO, reinforcing the regional and global relevance of this trend.

The epidemiological patterns observed during the study period are particularly concerning. Dengue transmission follows a cyclical and periodic behavior, with epidemic peaks occurring approximately every four to six years. This dynamic was clearly evidenced by the major national outbreaks recorded in 2014, 2019, and 2024, each dominated by a different viral serotype (DENV-1, DENV-2, and DENV-3, respectively), indicating a consistent alternation in viral circulation. These findings demonstrate that dengue transmission in Mexico follows predictable temporal and virological dynamics rather than stochastic epidemic emergence.

This periodicity shows strong synchronization with large-scale climatic drivers, particularly the ENSO. Epidemic peaks coincided with ENSO phases occurring during 2014–2016, 2018–2019, and 2023–2024. Recent evidence indicates that ENSO-related climatic variability can explain up to 63% of the interannual variation in global dengue incidence through climate teleconnections that modulate temperature, precipitation, and humidity patterns [77,78]. This association was further validated by a 40-year time-series analysis in Mexico, which demonstrated that multiyear dengue periodicity is largely driven by external climatic determinants rather than intrinsic epidemiological cycles [79]. Collectively, these findings suggest that anomalous warming during ENSO phases enhances *Aedes aegypti* vector competence, shortens the extrinsic incubation period, and amplifies the magnitude of cyclical epidemics.

Geographic heterogeneity further shapes dengue transmission across the country. Transmission remains concentrated in low-altitude coastal and inland corridors such as those in Jalisco and Oaxaca, where temperature and seasonal rainfall sustain vector populations and facilitate viral amplification [16,80]. In contrast, the recent intensification of dengue in arid Sonora is biologically plausible and increasingly documented. In desert settings, transmission can be sustained through microclimatic suitability during warm months, urban heat island effects that accelerate viral development, and anthropogenic water storage practices that generate persistent larval habitats despite the low ambient humidity [81,82,83]. These mechanisms explain why increases in Sonora differ ecologically from those observed in traditionally endemic coastal regions.

At the population level, dengue epidemiology in Mexico is modulated by a multifactorial interaction of social, environmental, and demographic determinants. Structural socioeconomic inequalities contribute to intermittent water supply and inadequate waste management, which act as major drivers of anthropogenic breeding sites and sustain high vector densities [84,85,86]. Housing precariousness increases human–vector contact, while population density and unplanned urbanization are strongly associated with the spatial distribution of dengue transmission [87,88]. Concurrently, climate change has facilitated the expansion of *Aedes aegypti* into higher-altitude regions, exposing immunologically naïve populations and reducing the viral extrinsic incubation period [89,90,91]. Additionally, migratory flows from hyperendemic regions, particularly along southern corridors, act as dynamic mechanisms for the introduction and dispersion of viral serotypes and genotypes, further complicating containment strategies [92,93,94].

The observed transition in dominant serotype from DENV-1 to DENV-2 and subsequently to DENV-3 reflects a critical reconfiguration of the national immunological landscape. This succession suggests that the reduction in individuals susceptible to previously circulating serotypes enables the establishment of new viral lineages through the exploitation of immunological niches. From a pathogenic perspective, this process increases the risk of severe dengue through ADE, whereby pre-existing non-neutralizing antibodies facilitate viral internalization into Fcγ receptor-bearing cells, leading to higher viral loads and exaggerated inflammatory responses [22,28,29].

This immunological mechanism, amplified by enhanced vector competence during extreme climatic conditions, likely contributed to the unprecedented magnitude and severity of the 2024 epidemic.

Beyond the current predominance of DENV-3, the epidemiological trajectory of Mexico suggests that the reemergence of DENV-4 may represent the next major public health challenge. Surveillance studies conducted between 2020 and 2025 reported an average DENV-4 prevalence of approximately 1.15%, with a peak prevalence of 2.1% during 2023 with 21,863 confirmed cases, without a clear association with clinical severity [79,95]. This historically low circulation has generated a substantial immunological susceptibility gap within the population. In a hyperendemic context where immunity against DENV-1, DENV-2, and DENV-3 is widespread, the reintroduction of DENV-4 could significantly elevate the risk of ADE and severe disease outcomes. Historical patterns of serotype replacement support the likelihood that the current dominance of DENV-3 may be succeeded by DENV-4 circulation [93], especially since the evolution of dengue virus in Mexico is characterized by frequent lineage replacement, positioning this serotype as a potential driver of the next epidemic cycle [96].

In this context of converging structural, climatic, immunological, and migratory pressures, vector control strategies alone are insufficient to curb dengue transmission. The implementation of second-generation tetravalent dengue vaccines with a short schedule, independent of serological screening, emerges as an essential complementary public health strategy capable of overcoming logistical barriers and mitigating disease burden in highly complex epidemiological settings.

Currently, dengue immunization in Mexico remains limited by regulatory and safety considerations. The CYD-TDV vaccine (Dengvaxia^®^, Sanofi Pasteur), although licensed, is not included in national immunization programs and is restricted by COFEPRIS to seropositive individuals aged 9–45 years due to the increased risk of severe disease among seronegative recipients [97]. Conversely, the tetravalent TAK-003 vaccine (Qdenga^®^, Takeda) is undergoing regulatory evaluation, representing a potential opportunity for broader population-level prevention, pending approval.

Collectively, the findings of this review highlight three defining features of dengue epidemiology in Mexico and Latin America: persistent cyclical epidemic patterns, recurrent alternation of dominant serotypes, and progressive intensification of outbreak magnitude. These dynamics have direct implications for public health planning, supporting anticipatory surveillance systems, integration of virological and genomic monitoring, targeted vaccine deployment aligned with epidemic cycles, and proactive allocation of resources. The coordinated implementation of surveillance, vaccination, and vector control strategies represents a feasible pathway to reduce dengue-related morbidity and mortality and strengthen long-term epidemic preparedness.

Several limitations should be considered when interpreting these findings. Dengue surveillance in Mexico is affected by underreporting due to national regulations that restrict molecular testing to approximately 10% of non-severe cases, resulting in underestimation of mild and asymptomatic infections. Additionally, heterogeneity in diagnostic capacity across states, updates in clinical classification systems, and potential disruptions to notifications during the COVID-19 pandemic may have influenced temporal trends. Nevertheless, the consistency of epidemiological patterns observed across multiple data sources and extended time periods supports the robustness of the principal conclusions.

## 12. Concluding Remarks

Dengue in Mexico exhibits a pattern of progressive intensification and predictable cyclicity. It is imperative to increase public resource allocation and strategically align these investments with projected epidemic cycles. Priority should be given to the enhancement of early warning systems during inter-epidemic periods, alongside the expedited regulatory clearance and implementation of vaccines, particularly in regions characterized by sustained transmission and low serotype-specific immunity. Coordinated integration of water infrastructure development, genomic surveillance, vector control, and immunization programs represents the most viable pathway toward achieving long-term reductions in both morbidity and mortality.

## Data Availability

Not applicable.
